# Protein elicitor PeVn1 induces resistance to *Botrytis cinerea* in strawberry and differential transcriptomic analysis

**DOI:** 10.3389/fmicb.2025.1541448

**Published:** 2025-05-13

**Authors:** Ziyu Zhang, Dong Wang, Yu Wang, Baozhu Dong, Jianxiu Hao, Hongyou Zhou

**Affiliations:** Key Laboratory of Biopesticide Creation and Resource Utilization for Autonomous Region Higher Education Institutions, College of Horticulture and Plant Protection, Inner Mongolia Agricultural University, Hohhot, China

**Keywords:** elicitors, *Fragaria × ananassa*, *Botrytis cinerea*, PeVn1, immunity, transcriptome

## Abstract

*Botrytis cinerea* is one of the most destructive diseases in strawberry cultivation. The protein elicitor PeVn1 is a secreted protein produced by *Verticillium nonalfalfae*, and it has been shown to enhance plant resistance against fungal infections. However, the mechanisms by which the protein elicitor acts remain poorly understood. In this study, we conducted physiological, biochemical, and transcriptomic analyses on strawberry leaves to reveal the resistance conferred by PeVn1 against *B. cinerea* infection. PeVn1 treatment significantly reduced lesion areas on *B. cinerea*-infected strawberry leaves. During the infection period, PeVn1 increased the activities of various antioxidant and defense-related enzymes, thereby enhancing the plant’s oxidative capacity. Compared to inoculation with *B. cinerea* alone, malondialdehyde (MDA) and electrical conductivity levels were significantly reduced. Transcriptomic analysis identified a total of 277 differentially expressed genes (DEGs) in the leaves treated with PeVn1 compared to the control group. The three most enriched KEGG pathways were the MAPK signaling pathway, plant hormone signal transduction, and plant-pathogen interaction, all of which are associated with plant immunity. DEGs associated with plant-pathogen interaction pathways included Calmodulin-like protein 1, Calcium-dependent protein kinase, and Chitin elicitor receptor kinase 1-like protein. DEGs linked to MAPK and hormone signaling pathways included EIN3-3, ethylene-responsive transcription factor 1, MAPK9 (MKK9), and transcription factor WRKY42. These genes play critical roles in PAMP-triggered immunity (PTI) and effector-triggered immunity (ETI). They support the plant’s hypersensitive response (HR), cell wall reinforcement, and other defense mechanisms. In summary, the protein elicitor PeVn1 activates the MAPK signaling pathway, increases calcium ions, and stimulates the ethylene signaling pathway in strawberry leaves, thereby enhancing plant resistance to infection. The results demonstrate that PeVn1 has significant potential to improve resistance against fungal diseases in strawberries.

## 1 Introduction

Strawberry (*Fragaria ananassa* Duch) belongs to the Rosaceae family and the genus *Fragaria*, is a perennial herbaceous plant known for its significant nutritional, medicinal, and health benefits (Fan et al., 2024). Gray mold disease, caused by *Botrytis cinerea*, is a major fungal infection that affects strawberries during both reproductive growth and post-harvest stages. This disease severely compromises fruit quality and shortens the market shelf life of strawberries. *B. cinerea* is a typical necrotrophic pathogen that rapidly kills host plant cells and tissues, utilizing nutrients from dead cells as a key component of its virulence strategy (van Kan, 2006). It also secretes toxic metabolites, including proteinaceous compounds and oxalic acid, which disrupt host plant interactions (Kars et al., 2005). During infection, *B. cinerea* penetrates the plant cuticle through lytic enzymes and phytotoxins, inducing reactive oxygen species (ROS) accumulation in host cell membranes. This process triggers oxidative bursts and subsequent cell death. Furthermore, the pathogen releases cell wall-degrading enzymes (CWDEs), such as pectinases, to disrupt epidermal cells. Pectin lyases and other proteins are also key virulence factors contributing to tissue maceration (Nafisi et al., 2014). Collectively, these mechanisms ensure nutrient availability for fungal growth (Deighton et al., 2001).

Plants have evolved multiple defense strategies to combat *B. cinerea* infection. During early infection stages, microbe-associated molecular patterns (MAMPs) and damage-associated molecular patterns (DAMPs) activate plant pattern recognition receptors (PRRs), triggering early immune responses (Newman et al., 2013). For instance, the host plasma membrane receptor LYM2 recognizes the MAMP chitin, initiating an immune cascade (Miya et al., 2007; Faulkner et al., 2013). DAMPs, endogenous elicitors released by damaged plant tissues during pathogen invasion or abiotic stress, include oligosaccharides (OGs) derived from the cell wall. These OGs function as DAMPs to enhance resistance against *B. cinerea* (Ferrari et al., 2013). Additionally, plant polygalacturonase-inhibiting proteins (PGIPs) inhibit pectin-degrading enzymes (endoPGs). Overexpression of PGIPs in *Arabidopsis* suppresses *B. cinerea*-secreted pectinase activity, bolstering plant defenses (De Lorenzo et al., 2011). Brassinosteroid Insensitive 1-Associated Kinase 1 (BAK1) plays a critical role in immunity against necrotrophic pathogens (Zhang et al., 2013). Botrytis-induced kinase 1 (BIK1), a receptor-like cytoplasmic kinase (RLCK), is essential for resistance to *B. cinerea*. Research demonstrates that *Arabidopsis bik1* mutants exhibit heightened susceptibility to necrotrophic pathogens (Veronese et al., 2006). During later infection stages, the plant immune system elevates MPK4 activity. Within the MPK4-WRKY33-MKS1 complex, phosphorylated MKS1 releases WRKY33, thereby upregulating *PAD3* expression (Qiu et al., 2008). Furthermore, MAPK4 and MAPK6 (mitogen-activated protein kinases 4 and 6) modulate ethylene signaling via phosphorylation of ERF104, enhancing resistance to *B. cinerea* (Bethke et al., 2009).

It is evident that plants and the microorganisms in their vicinity have evolved in a manner that facilitates the recognition of MAMPs by the plant itself, either in the microorganisms or in their derivatives (Li et al., 2023). The binding of MAMPs to PRRs initiates signal transduction cascades that enhance plant resistance against pathogenic bacteria (Vlot et al., 2021). Elicitors activate diverse defense responses in host plants: First, PRR recognition triggers stomatal closure to restrict pathogen entry; Second, these immune receptors transduce immune signals through Ca^2+^ fluxes, ROS, G-proteins, and MAPK cascades to activate downstream defense responses, including early immune events such as callose deposition, protein phosphorylation, or activation of plasma membrane proteins. These responses are amplified through transcriptional and metabolic reprogramming, leading to physiological adaptations such as cell wall reinforcement, antimicrobial metabolite production, pathogenesis-related (PR) protein synthesis, and activation of oxidative stress-protective enzymes (Schwessinger and Ronald, 2012). The protein elicitor HrpN has been demonstrated to induce resistance in *N. benthamiana* to *B. cinerea*, while concomitantly promoting plant growth and development (Miao et al., 2010; Lawaju et al., 2018). The *Verticillium dahliae* Aspf2-like protein (VDAL) induces broad-spectrum fungal resistance in plants, enhancing Brassica resistance to *Sclerotinia sclerotiorum* while activating defense-related enzymes and upregulating MAPK signaling pathway genes (Jiang et al., 2022). PevD1 protein triggers calcium-mediated signaling cascades by binding specific domains of NR2 protein, ultimately inducing systemic acquired resistance (Liang et al., 2021). BAR11 elicitor interacts with *Arabidopsis* peroxidases to modulate H₂O₂ levels, thereby enhancing pathogen resistance (Wang et al., 2024). Various protein elicitors have been identified in biocontrol fungi and bacteria (Freitas et al., 2014; Gaderer et al., 2015; Gomes et al., 2015), all capable of inducing systemic acquired resistance in plants. Notably, harzianolide from *Trichoderma harzianum* upregulates defense-related enzymes and genes, playing crucial roles in both plant immunity and growth regulation (Cai et al., 2013). Collectively, these findings demonstrate that protein elicitors significantly enhance plant disease resistance through multifaceted mechanisms.

The protein elicitor PeVn1 is a novel elicitor isolated in our laboratory from the fermentation liquid of *Verticillium nonalfalfae*. Previous studies indicate that PeVn1 triggers reactive oxygen species (ROS) burst, callose deposition, mitogen-activated protein kinase (MAPK) activation, and accumulation of defense-related metabolites. It also induces necrotic lesions resembling a hypersensitive response (HR) and upregulates defense-related genes. Notably, PeVn1 enhances plant resistance to fungal and bacterial pathogens, as well as Tobacco Mosaic Virus expressing green fluorescent protein (TMV-GFP) (Zhang et al., 2024). Despite its broad-spectrum antifungal activity, the potential of PeVn1 to confer resistance against *B. cinerea* in strawberry plants remains uncharacterized. In this study, we integrated physiological, biochemical, and transcriptomic approaches to dissect PeVn1-induced immune responses in strawberry plants. This study aims to unravel the molecular mechanisms underlying *B. cinerea* resistance, providing novel insights for sustainable disease management strategies.

## 2 Materials and methods

### 2.1 Plant and strain culture conditions and experimental treatments

In this experiment, strawberry leaves of the “Benihoppe” variety were utilized as the plant material. The plants were cultivated under a 16 h light/8 h dark cycle at 25°C/22°C. The protein elicitor PeVn1 was prepared at a concentration of 500 μg/mL immediately before use, ensuring that all solutions were freshly prepared. Strawberry leaves were treated with 500 μg/mL PeVn1 or PBS buffer (Control) via foliar spray.

*Botrytis cinerea* was stored at −80°C and reactivated on Potato Dextrose Agar (PDA) medium. Five millimeter puncher was employed to transfer fungal mycelium to the center of the agar plates, which were then incubated at 25°C for 3 days. Freshly collected mycelium was treated with PeVn1 for 24 h and subsequently transferred onto the surface of strawberry leaves, which were maintained in a humidity controlled chamber at 90% relative humidity.

The strawberry leaves were treated with the protein elicitor PeVn1, and 48 h later, they were inoculated with *B. cinerea*. The leaves were harvested at 0, 6, 12, and 24 h, respectively, after the inoculation. Lesion areas were quantified using ImageJ software. The infected leaves were immediately frozen in liquid nitrogen and stored at −80°C for subsequent analysis. Each experiment included three independent biological replicates.

### 2.2 *In vitro* test of *B. cinerea* on PDA medium sprayed with PeVn1

PeVn1 (500 μg/mL) and PBS buffer solution (Control) were uniformly applied to the PDA medium. Following treatment *B. cinerea* was inoculated at the center of the medium using a 5 mm puncher. The petri dish was subsequently incubated at a constant temperature of 25°C. The growth of *B. cinerea* in the petri dish was monitored at 6, 12, and 36 h.

### 2.3 Enzyme activity assay

The activities of superoxide dismutase (SOD), peroxidase (POD), and polyphenol oxidase (PPO) were determined using the kits YX-C-A500, YX-C-A502, and YX-C-404 (Shanghai Youxuan Biotechnology Co., Ltd., Shanghai, China) respectively. Similarly the activities of catalase (CAT), *β*-1,3-glucanase and phenylalanine ammonia-lyase (PAL) were measured using the kits G0105F, G0526F, and G0114F (Suzhou Gerei Biotechnology Co., Ltd., Suzhou, China), respectively. Enzyme activities were assayed following the manufacturer’s instructions (Zhang et al., 2020).

### 2.4 Determination of MDA, soluble sugar content, and relative electroconductivity

Soluble sugar content was determined using the G501F kit (Suzhou Gerei Biotechnology Co., Ltd., Suzhou, China). Soluble sugar content (mg·g^−1^ FW) was calculated as 0.41 × (A450-0.0203)/W. Malondialdehyde (MDA) content was determined as follows (Zhang et al., 2023): 0.1 g of plant leaves was mixed with 1 mL of 10% trichloroacetic acid (TCA) and homogenized in an ice bath. The homogenate was centrifuged at 12,000 rpm for 10 min at 4°C. The resulting supernatant was collected as the sample. One milliliter of 0.6% thiobarbituric acid was added to 1 mL of extract, mixed thoroughly, and incubated at 95°C for 30 min. After rapid cooling, the mixture was centrifuged at 12,000 rpm for 10 min at 25°C. The absorbance was measured at 532 and 600 nm. MDA content (nmol·g^−1^ FW) was calculated as 16.1 × (A532-A600)/W, where A532 and A600 are absorbances at 532 and 600 nm, respectively, and W represents the fresh weight of the plant tissue. Relative electrolyte leakage was measured as follows (Yang et al., 2021): Leaves were washed with deionized water, punched into circular disks without main veins, and placed in test tubes containing 10 mL of deionized water, each tube holding 10 disks. The tubes were incubated in a vacuum desiccator for 30 min and subsequently shaken for 1 h. Initial conductivity (S1) was measured using a conductivity meter. The tubes were then placed in a boiling water bath for 10 min to release all cellular contents, cooled to room temperature, and the final conductivity (S2) was measured. Relative leakage was calculated using the formula: Relative leakage = S1/S2.

### 2.5 Transcriptomics analysis

Strawberry leaves were treated with 500 μg/mL PeVn1 via foliar spray, while PBS-treated plants served as the control. After 24 h, three leaves per group (three biological replicates) were collected, transferred to 1.5 mL centrifuge tubes, and flash-frozen in liquid nitrogen. RNA extraction, library construction, and sequencing were conducted by Wuhan Kangce Technology Co., Ltd. (Wuhan, China). Raw sequencing data were preprocessed using fastp (version 0.23.2) to remove low-quality reads and trim adapters. Clean reads were then processed with in-house scripts to mitigate PCR and sequencing biases. Briefly, reads were clustered by unique molecular identifier (UMI) sequences, and pairwise alignment was performed within each cluster. Sub-clusters with >95% sequence identity were retained, followed by multiple sequence alignment to generate consensus sequences. Collectively, these steps minimized technical artifacts from library preparation and sequencing.

The resulting sequences were subjected to standard mRNA-seq analysis. Sequences were mapped to the reference genome using STAR software (v2.7.6a). Reads mapping to exon regions were counted using featureCounts (Subread v1.5.1; Bioconductor), and RPKM values were calculated. Differentially expressed genes (DEGs) between groups were identified using the edgeR package (v3.40.2) with a *p*-value cutoff of 0.05 and a fold-change threshold of 2. Gene Ontology (GO) and Kyoto Encyclopedia of Genes and Genomes (KEGG) enrichment analyses for DEGs were performed using KOBAS software (v2.1.1) with a p-value cutoff of 0.05 to identify statistically significant enrichments. Gene Set Enrichment Analysis (GSEA) was conducted using GSEA software with GO and KEGG background datasets from the MSigDB database.

### 2.6 RNA extraction and RT-PCR analysis

Total RNA was extracted from strawberries leaf blades using the Plant RNA Extraction Kit (TAKARA). cDNA was synthesized by reverse transcription using PrimeScript™ RT Master Mix (TAKARA). Quantitative PCR (qPCR) was conducted using the LightCycler 96 System (Roche) and TB Green^®^ Premix Ex Taq™ II (TAKARA) to assess gene expression levels. Each experiment included three biological and three technical replicates, and the *EF1α* gene strawberries leaf served as a reference gene for normalization. Relative gene expression was calculated using the 2^–ΔΔCt^ method (Livak and Schmittgen, 2001). All primers used for RT-PCR are listed in Supplementary Table 5.

### 2.7 Statistical analysis

All the data presented are the mean values of three biological repetitions and were statistically analyzed via one-way ANOVA and Tukey’s test (*p < 0.05; **p < 0.01; ***p < 0.001;****p < 0.0001) using GraphPad Prism 8.0 software.

## 3 Results

### 3.1 Determination of PeVn1-induced disease resistance of strawberry to *B. cinerea*

**Figure 1:**
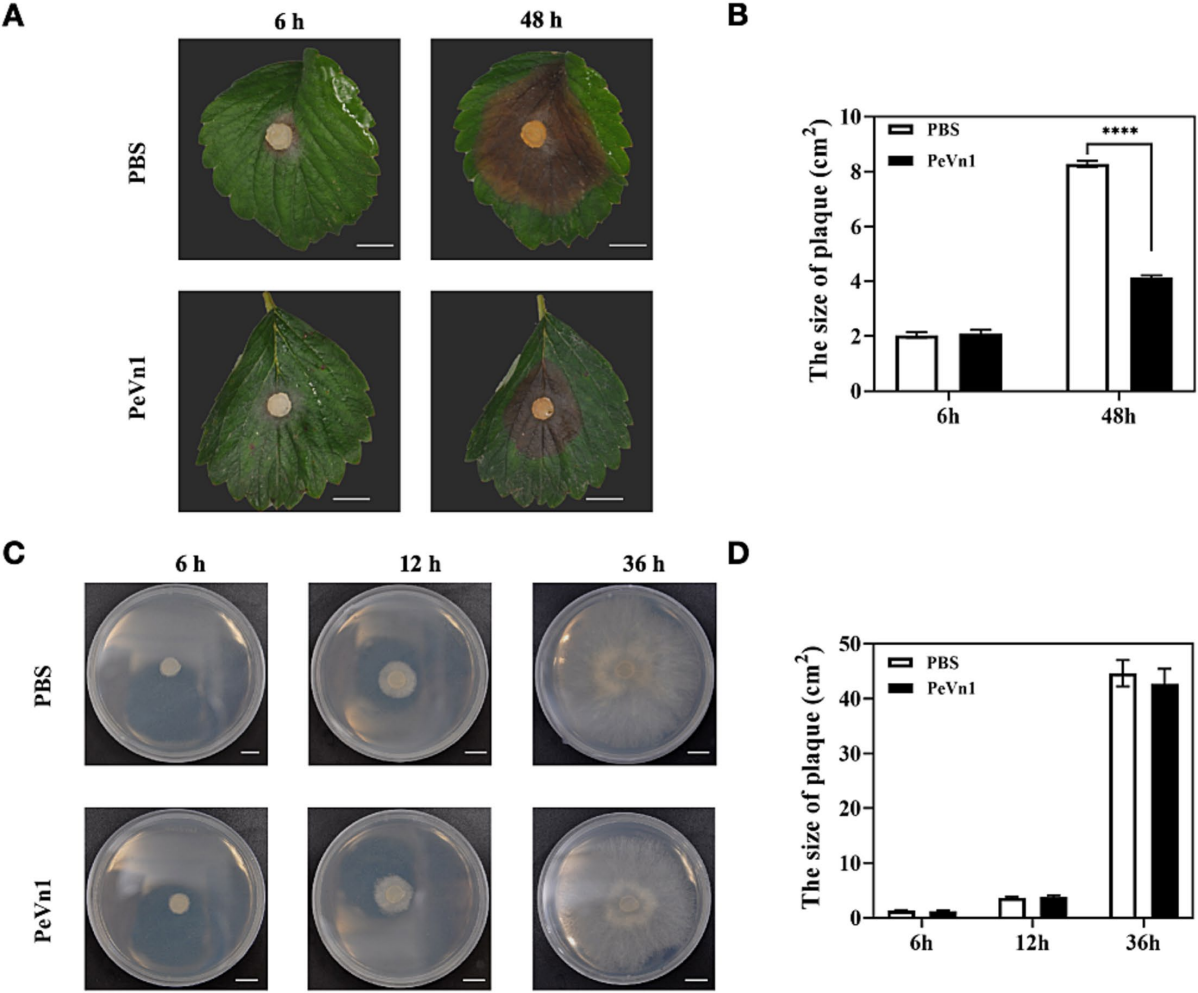
Effect of the protein elicitor PeVn1 on *B. cinerea* resistance. **(A)** Morphology and phenotype of PeVn1 and PBS-treated leaves inoculated with *B. cinerea.* **(B)** Histogram of spot area of strawberry leaves inoculated with *B. cinerea* after PeVn1 and PBS treatments. A total of 10 independent replications were performed. **(C)** Growth of *B. cinerea* on PDA medium after inoculation with PeVn1 and PBS for 6, 12, and 36 h, respectively. **(D)** Colony area of *B. cinerea* on PDA medium after treatment with PeVn1 and PBS. Three independent biological replicates were performed. Student’s *t*-test (****p < 0.0001) was used to estimate significant effects. Bars are 1 cm.

To investigate the effect of the protein elicitor PeVn1 on *B. cinerea* resistance, strawberries were sprayed with PeVn1 and incubated for 48 h before *B. cinerea* inoculation. PeVn1 significantly enhanced the immune response of strawberries against *B. cinerea*, reducing the lesion area by 49.98% compared to the control group (Figures 1A,B). To assess whether PeVn1 directly inhibits *B. cinerea* growth, fungal colonies were cultured on PDA medium supplemented with PeVn1. No significant difference in fungal growth was observed after 36 h (Figures 1C,D). These results suggest that PeVn1 does not directly inhibit *B. cinerea* but activates the immune response of strawberries.

### 3.2 Antioxidant enzyme activity assay

**Figure 2:**
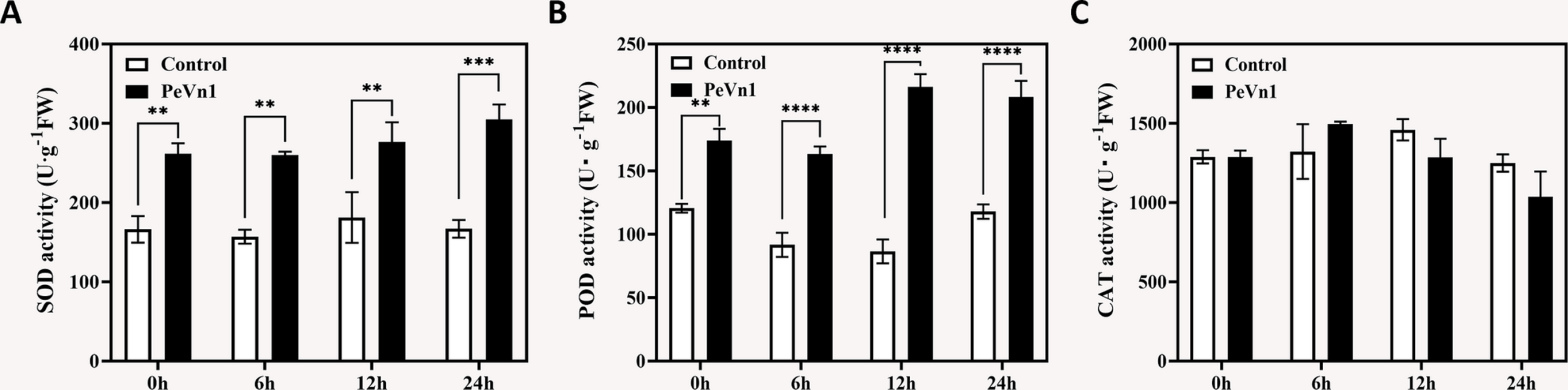
Antioxidant enzyme activities in strawberries inoculated with *B. cinerea* following treatment with PBS or 500 μg·mL^−1^ PeVn1. **(A)** SOD, **(B)** POD, **(C)** CAT activity in *F. × ananassa* at 0, 6, 12, and 24 h post-inoculation. Student’s *t*-test (**p < 0.01; ***p < 0.001; ****p < 0.0001) was used to estimate significant effects.

Plants modulate antioxidant enzyme activities and reactive oxygen species (ROS) homeostasis to cope with biotic stress (Parida et al., 2004). We quantified three key antioxidant enzymes in strawberry leaves following PeVn1 treatment. Superoxide dismutase (SOD) activity in PeVn1-treated leaves increased progressively, peaking at 24 h with an 82.74% elevation compared to the control (Figure 2A). Similarly, peroxidase (POD) activity was significantly higher at all timepoints, showing 57.34, 65.77, and 52.62% increases at 6, 12, and 24 h, respectively (Figure 2B). In contrast, catalase (CAT) activity remained stable in control plants but exhibited a transient 15% reduction at 12 h post-treatment in PeVn1-treated leaves (Figure 2C). These results indicate that PeVn1 selectively enhances SOD and POD activities, which are critical for H_2_O_2_ detoxification and lignin polymerization. This targeted antioxidant response may strengthen cell wall integrity and limit *B. cinerea* colonization.

### 3.3 Assays of general defense-related enzyme activities

**Figure 3:**
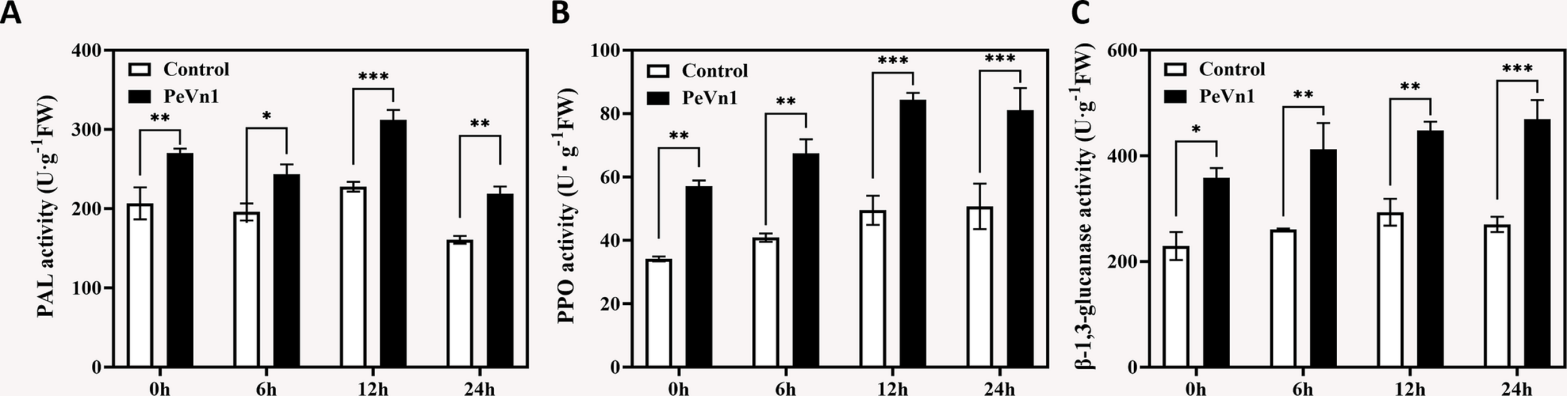
Defense-related enzyme activities in strawberry leaves inoculated with *B. cinerea* following treatment with PBS or 500 μg·mL^−1^ PeVn1. **(A)** PAL, **(B)** PPO, **(C)** β-1,3-glucanase activity in *F. × ananassa* at 0, 6, 12, and 24 h post-inoculation. Student’s *t*-test (*p < 0.05; **p < 0.01; ***p < 0.001) was used to assess statistical significance.

Polyphenol oxidase (PPO), phenylalanine ammonia-lyase (PAL), and *β*-1,3-glucanase are pivotal enzymes in plant defense against pathogens (Wang et al., 2012). We quantified their activities in strawberry leaves following PeVn1 treatment. PAL activity surged by 68% at 6 h and peaked at 12 h with a 2.1-fold increase compared to controls (Figure 3A). PPO exhibited a parallel trend, reaching maximal induction (1.7 fold) at 12 h (Figure 3B). β-1,3-glucanase activity showed progressive enhancement, culminating in a 73.79% increase at 24 h (Figure 3C). Control plants displayed stable enzyme levels across all timepoints. This indicates that phenylalanine ammonia-lyase (PAL), polyphenol oxidase (PPO), and β-1,3-glucanase all play a synergistic activating role in the immune response of strawberry leaves induced by the protein elicitor PeVn1.

### 3.4 Assays of MDA, relative conductivity, and soluble sugar content

**Figure 4:**
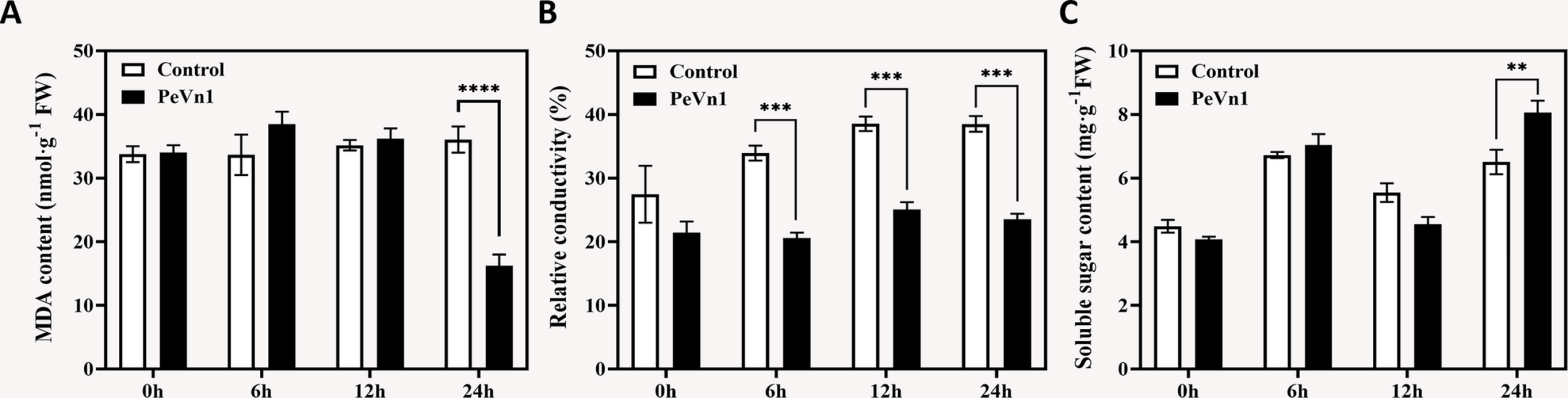
Injury-related indicators in strawberries inoculated with *B. cinerea* following treatment with PBS or 500 μg·mL^−1^ PeVn1. **(A)** MDA, **(B)** Relative conductivity, **(C)** Soluble sugar content in *F. × ananassa* at 0, 6, 12, and 24 h post-inoculation. Three independent replicates were performed for MDA and soluble sugar content. Six independent replicates were used for relative conductivity. Student’s *t*-test (**p < 0.01; ***p < 0.001; ****p < 0.0001) was used to assess statistical significance.

Relative conductivity and MDA content are widely recognized indicators of membrane stability, reflecting the extent of cell membrane damage and oxidative stress (Carter et al., 2007). We further assessed the antioxidant capacity of strawberries treated with the protein elicitor PeVn1. The results showed that the MDA content remained relatively stable over time after treatment with the protein elicitor PeVn1, but exhibited a significant decrease at 24 h, reaching its lowest value (Figure 4A). During the 12 h infection period, the relative electrical conductivity gradually increased in both treatments. The relative electrical conductivity of the control consistently exhibited significantly higher values compared to the PeVn1-treated samples after 6 h of infection (Figure 4B). This suggests that PeVn1 could effectively reduce the extent of cell membrane damage caused by *B. cinerea*.

The increased soluble sugar content serves as an indicator of the plant capacity to activate an immune response against fungal pathogens (Jeandet et al., 2022). We measured the soluble sugar content in both treatment groups, observing significant differences at 24 h. The reason behind the gradual increase in soluble sugar content at 6 h followed by a decline at 12 h remains unclear (Figure 4C). Overall, the immune response induced by PeVn1 in strawberry leaves may not increase the content of sugars related to the expression of defense genes against *B. cinerea*.

### 3.5 Transcriptome changes in response to PeVn1

To investigate the molecular response associated with PeVn1-induced resistance to *B. cinerea*, transcriptome profiles of strawberries were analyzed 24 h post-inoculation. RNA-Seq data from the Control and PeVn1 treatment groups, each consisting of three biological replicates, were analyzed (Supplementary Table 1). The base error rate was low at 0.01%, with Q20 values exceeding 97.46% and Q30 values surpassing 93.89%, while the GC content ranged from 47.61 to 47.75%.

**Figure 5:**
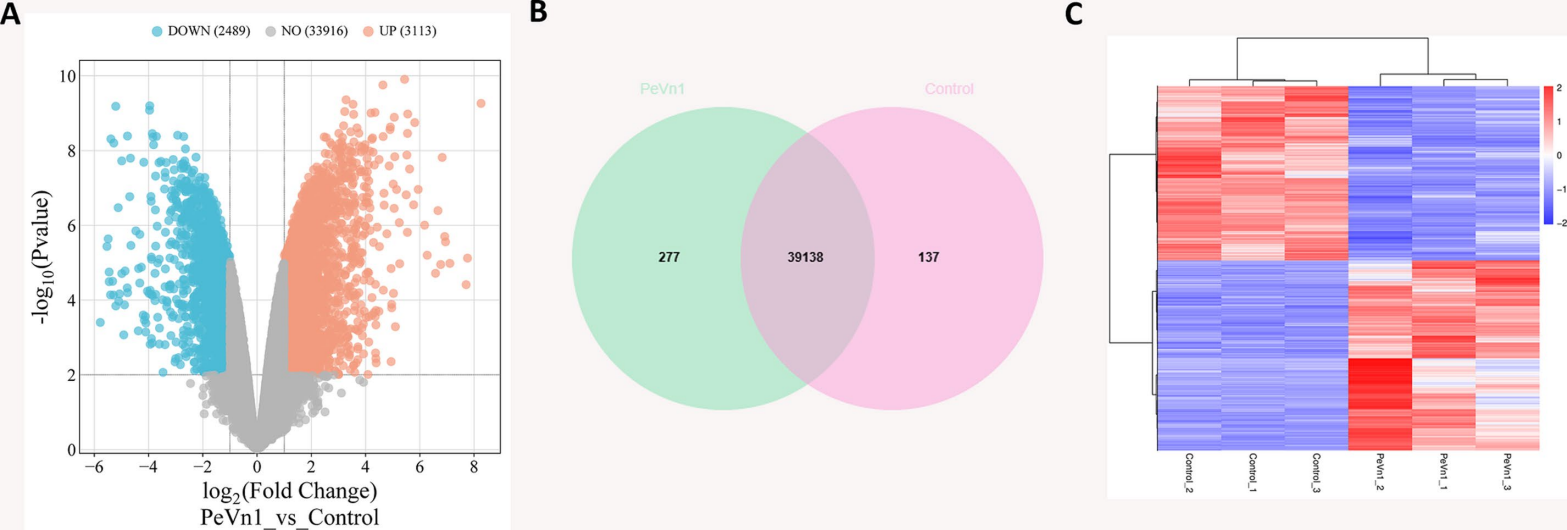
Analysis of PeVn1-induced differentially expressed genes in strawberry. **(A)** Volcano plot showing the distribution of overall DEGs. The sum of read counts for each gene across different samples in the volcano plot is greater than one. Genes with significant differences between the two groups were selected based on the thresholds |log2 Fold Change| ≥ 1 and Padj ≤ 0.01. The X-axis represents fold change in gene expression, and the Y-axis represents statistical significance. Orange dots indicate significantly up-regulated DEGs (UP), blue dots represent significantly down-regulated DEGs (DOWN), and gray dots represent non-differentially expressed genes (NO). **(B)** Venn diagram illustrating the statistics for all expressed genes with RPKM > 1 in both PeVn1 and Control samples. The green area represents genes expressed in the PeVn1-treated group, the pink area represents genes expressed in the Control group, and the overlapping area shows the genes co-expressed in both groups. **(C)** Heatmap of DEGs.

All clean reads were subsequently *de novo* assembled using StringTie (v1.3.3b), and the assembly results were assessed. A total of 255,112,770 megabases (Mb) of reads were obtained across six samples, with an average of 42,518,795 Mb reads per sample (Supplementary Table 2). A total of 39,518 genes exhibited Reads per Kilobase per Million Reads (RPKM) values greater than one in both the PeVn1 and Control groups. Compared to the control group, PeVn1 treatment resulted in significant upregulation of 2,489 genes and downregulation of 3,113 genes (Figure 5A). Among these genes, 39,138 were co-expressed in both groups, 277 were uniquely expressed in the PeVn1 group, and 137 were only present in the Control group (Figure 5B). Heatmap clustering analysis of differentially expressed genes (DEGs) demonstrated consistent gene expression trends within replicate groups, while significant differences were observed between the groups (Figure 5C).

### 3.6 Gene Ontology and Kyoto Encyclopedia of Genes and Genomes pathway analysis

**Figure 6:**
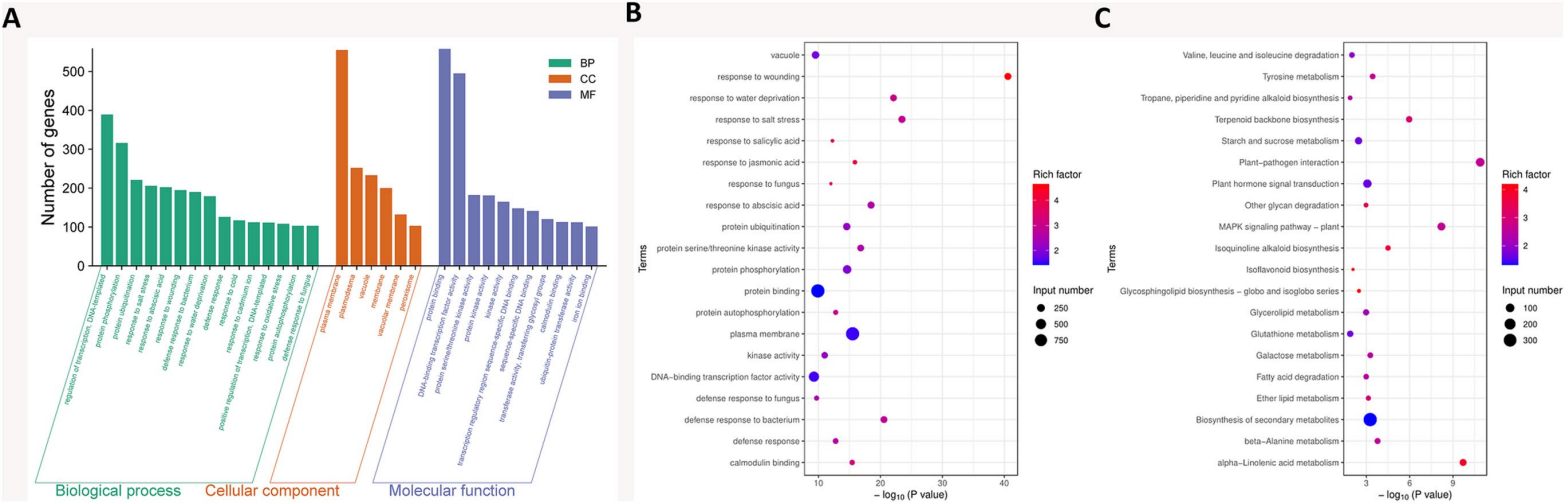
**(A)** GO enrichment analysis of DEGs. The bar graphs display the 32 most significantly enriched GO terms (ranked by adjusted *p*-values), along with the numbers of up- and down-regulated genes corresponding to the top 10 GO terms across three categories: BP (biological process), MF (molecular function), and CC (cellular component). **(B)** ScatterplotofGOenrichmentanalysi. **(C)** Bubble chart illustrating the KEGG classification of DEGs. The KEGG dot plot shows the top 20 categories, ranked by the proportion of genes in each category. The size of each dot, represented by “Count,” indicates the number of genes associated with the significant DEGs list for that KEGG term, while the color of the dots reflects the adjusted *p*-values. The gene ratio represents the proportion of differentially expressed genes annotated to a particular KEGG pathway relative to the total number of DEGs. Dot size indicates the number of genes annotated to the KEGG pathway, with the color gradient from red to purple representing the degree of enrichment.

GO functional enrichment analysis of 5,741 DEGs between the PeVn1 and Control groups identified 263 GO terms, including 162 biological processes (BP), 82 molecular functions (MF), and 19 cellular components (CC), which accounted for 61.59, 31.18, and 7.2%, respectively (Supplementary Table 3). The top 34 most significant GO terms across the three categories (BP, MF, and CC) were selected for further classification and analysis (Figure 6A). The 10 most significantly enriched BP terms were: “regulation of transcription, DNA-templated” (GO:0006355), “protein phosphorylation” (GO:0006468), “protein ubiquitination” (GO:0016567), “response to salt stress” (GO:0009651), “response to abscisic acid” (GO:0009737), “response to wounding” (GO:0009611), “defense response to bacterium” (GO:0042742), “response to water deprivation” (GO:0009414), “defense response” (GO:0006952), and “response to cold” (GO:0009409). The 10 most significantly enriched MF terms were: “protein binding” (GO:0005515), “DNA-binding transcription factor activity” (GO:0003700), “protein serine/threonine kinase activity” (GO:0004674), “protein kinase activity” (GO:0004672), “kinase activity” (GO:0016301), “transcription regulatory region sequence-specific DNA binding” (GO:0000976), “sequence-specific DNA binding” (GO:0043565), “transferase activity, transferring glycosyl groups” (GO:0016757), “calmodulin binding” (GO:0005516), and “ubiquitin-protein transferase activity” (GO:0004842). In the cellular component (CC) category, the most significantly enriched terms were “plasma membrane” (GO:0005886) and “membrane” (GO:0016020) (Figure 6B).

KEGG pathway analysis offers comprehensive pathway information that aids in understanding the systemic biological functions of genes, including metabolic pathways, genetic information processing, and various cellular processes. This analysis provides valuable insights into how the protein elicitor PeVn1 activates the immune system in strawberries to defend against *B. cinerea*. The enrichment analysis of DEGs in the KEGG database identified a total of 20 pathways. Among these, 11 pathways contained a higher number of upregulated genes, while 9 pathways contained more downregulated genes (Figure 6C). The three most significantly enriched pathways (Padj < 0.05) were: “Plant-pathogen interaction” (fve04626), “alpha-Linolenic acid metabolism” (fve00592), and “MAPK signaling pathway - plant” (fve04016) (Supplementary Table 4). Within these pathways, 101 genes were associated with plant-pathogen interactions, 7 genes were part of the MAPK signaling pathway, and 91 genes were involved in plant hormone signaling. These findings suggest that PeVn1 activates early signal transduction mechanisms, which play a crucial role in enhancing the plant’s resistance to *B. cinerea*, highlighting the importance of both pathogen recognition and defense response pathways.

### 3.7 Gene expression analysis of disease resistance-related pathways

**Figure 7:**
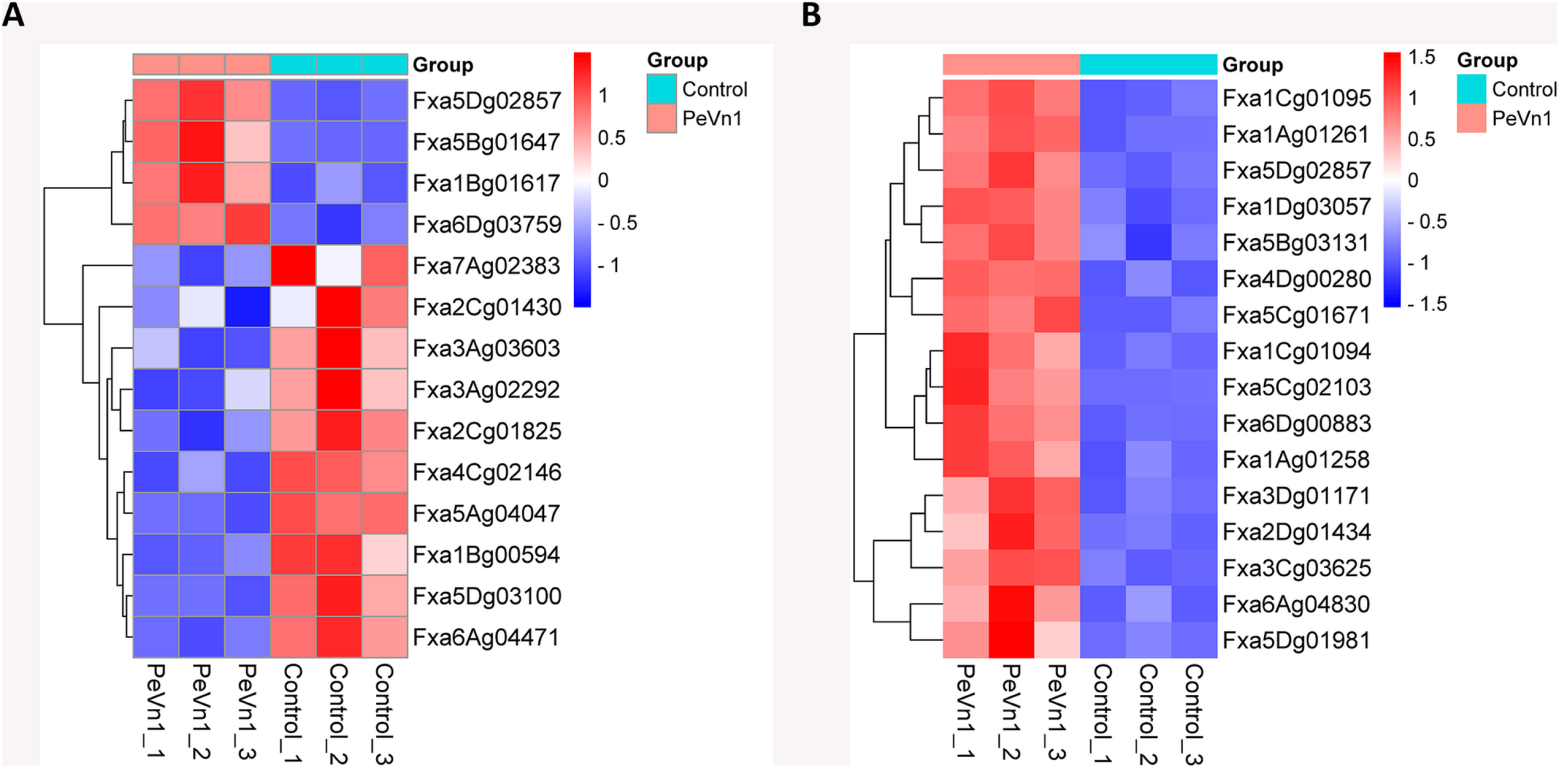
Hierarchical clustering heatmap of gene expression in strawberry plants treated with PBS or PeVn1 and inoculated with *B. cinerea*. **(A)** Heatmap of genes involved in MAPK signaling and plant-pathogen interactions. **(B)** Heatmap of genes related to MAPK signaling and plant hormone signal transduction. The horizontal axis represents the sample names, while the vertical axis shows the normalized RPKM values of differentially expressed genes. Redder hues indicate higher expression levels, while bluer hues indicate lower expression levels.

The mechanism by which the protein elicitor PeVn1 induces disease resistance involves coordinated activation of multiple signaling pathways. GO and KEGG enrichment analyses demonstrated that numerous DEGs are enriched in the plant-pathogen interaction, MAPK, and plant hormone signaling pathways. The most enriched DEGs from these three pathways were selected for subsequent heatmap analysis. Among the 14 genes, Fxa5Dg02857 (BRI1-associated receptor kinase), Fxa5Bg01647 (Calcium-dependent protein kinase), Fxa1Bg01617 (Calmodulin-like protein 1), and Fxa6Dg03759 (Chitin elicitor receptor kinase 1-like protein) were found to simultaneously regulate both the MAPK and plant-pathogen interaction pathways, and were upregulated in response to PeVn1 treatment (Figure 7A). Other genes that were downregulated include jasmonic acid-amido synthetase, serine/threonine-protein kinase, protein phosphatase 2C, and transcription factor TGA7. In contrast, 16 genes linked to the MAPK and plant hormone signaling pathways (Figure 7B) were all upregulated. These findings suggest that PeVn1 activates calcium sensor genes, which subsequently stimulate downstream MAPK and plant hormone signaling pathways, thereby initiating the plant’s immune response.

### 3.8 Verification of differential expression by quantitative RT-PCR

**Figure 8:**
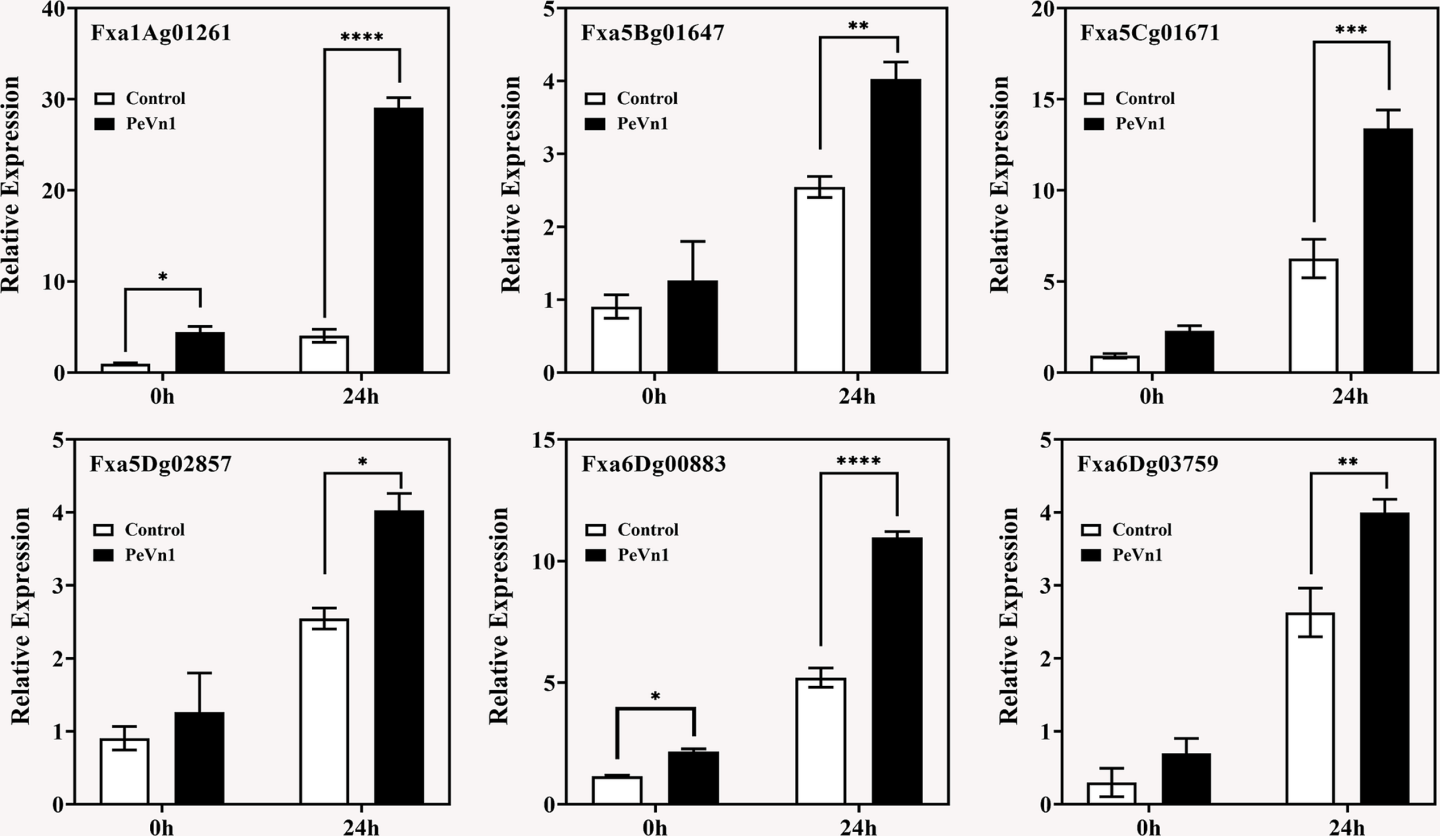
Expression levels of strawberry defense-related genes against *B. cinerea* at 0 and 24 h post-inoculation, following treatment with PBS or PeVn1, relative to the control. Student’s *t*-test (*p < 0.05; **p < 0.01; ***p < 0.001; ****p < 0.0001) was used to assess statistical significance.

To validate the transcriptomic data and elucidate the mechanism by which PeVn1 induces plant resistance, DEGs associated with disease resistance from the KEGG pathways (including Fxa6Dg00883, Fxa5Cg01671, Fxa1Ag01261, Fxa5Dg02857, Fxa5Bg01647, and Fxa6Dg03759) were selected for fluorescence quantitative PCR verification (Figure 8). These DEGs exhibited significant upregulation in the transcriptomic analysis. Consistent with expectations, the quantitative PCR results demonstrated that, in the PeVn1 treatment, the relative expression levels of most DEGs were elevated compared to the control group. This independently validated the reliability of the high coverage obtained in the transcriptomic data.

## 4 Discussion

Plant immune elicitors are recognized by receptors on the surface of plant cells, which then activate the plant’s intricate and robust innate immune system, initiating defensive responses to minimize pathogen damage (Wang et al., 2020; Sun et al., 2022). Due to the conservation of foundational resistance responses across plant species, the basic mechanisms through which elicitors activate plant immunity are largely similar. These mechanisms typically involve global, multi-site, and multi-pathway regulatory cascades, which enhance plant resistance by triggering a paradigmatic series of signaling events (Kanyuka and Rudd, 2019). Such signaling events activate microbe/pathogen-associated molecular patterns (M/PAMPs), initiating either pattern-triggered immunity (PTI), effector-triggered immunity (ETI), or both PTI and ETI simultaneously. These responses collectively aim to directly or indirectly suppress pathogen growth and reproduction (Nishad et al., 2020).

In plant cells the production and removal of reactive oxygen species (ROS) are maintained in a dynamic balance. However, during defense responses, ROS metabolism can become disrupted, leading to the accumulation of excessive ROS within cells. This accumulation increases cell membrane permeability, and in severe cases, results in cell death (Garcia-Brugger et al., 2006). Upon pathogen infection or elicitor treatment, the activation of protective enzymes associated with disease resistance constitutes a crucial mechanism for inducing plant immunity. Key enzymes, including catalase (CAT), superoxide dismutase (SOD), peroxidase (POD), phenylalanine ammonia lyase (PAL), polyphenol oxidase (PPO), and *β*-1,3-glucanase, are integral to plant defense against pathogen invasion (Apel and Hirt, 2004). Their activity levels are often used as reliable indicators of plant defense responses. Notably, β-1,3-glucanase is involved in the salicylic acid (SA) and jasmonic acid (JA) defense pathways (Fukamizo and Shinya, 2019; Wang et al., 2021). In this study, strawberry leaves treated with PeVn1 exhibited enhanced antioxidant capacity, with significantly elevated activities of SOD, POD, PAL, PPO, and β-1,3-glucanase compared to the control group, whereas no significant difference was observed in CAT activity (Figure 2). Furthermore, after 12 h of *B. cinerea* infection, CAT activity showed a decreasing trend. This decrease may be attributed to the requirement of CAT activity to interact with intracellular calcium-binding proteins, SA, and nitric oxide (NO), both of which inhibit CAT activity (Chen et al., 1993; Durner and Klessig, 1996). Excessive ROS accumulation also compromises cell membrane integrity, leading to the leakage of electrolytes and other substances, thereby disrupting cellular osmotic balance. To mitigate these effects, plants generate osmotic regulators, such as soluble sugars, which aid in self-regulation of osmotic pressure. Malondialdehyde (MDA) content serves as a key marker for oxidative stress levels. In our study, in strawberry leaves treated with PeVn1, the relative conductivity and MDA content significantly decreased after 24 h, while soluble sugar content increased significantly compared to the control group. These findings suggest that the protein elicitor PeVn1 reduces MDA accumulation, decreases H_2_O_2_ and O_2_^−^ influx into cells, and enhances antioxidant capacity, thereby strengthening plant resistance against *B. cinerea*. In summary, the protein elicitor PeVn1 stimulates antioxidant responses and defensive enzyme activities in strawberry leaves, thereby enhancing resistance against *B. cinerea* infection and activating robust plant defense mechanisms.

Plant immune elicitors trigger extracellular Ca^2+^ influx by binding to cell surface receptors, leading to increased intracellular Ca^2+^ concentrations. The binding of Ca^2+^ to the EF-hand domain of calmodulin activates pathogenesis-related proteins and resistance transcription factors (Yang and Poovaiah, 2003). Transcriptomic data from this study demonstrate that the PeVn1-induced resistance mechanism against *B. cinerea* in strawberry involves synergistic regulation of multiple pathways. GO and KEGG enrichment analyses revealed that numerous differentially expressed genes were enriched in pathways including MAPK signaling, plant hormone signaling, and plant-pathogen interaction. Within the calcium signaling-MAPK cascade pathway, PeVn1 significantly upregulated calcium-dependent protein kinase (CDPK, Fxa5Bg01647) and calmodulin-like protein (CML1, Fxa1Bg01617), activating downstream MAPK components MKK9 and MPK3/6, which subsequently induced the expression of ERF1 (Figure 7A), a key transcription factor in the ethylene signaling pathway. This cascade exhibits high conservation with the MAPK-ethylene (ET) synergistic resistance mechanism in *Arabidopsis* (Xu and Zhang, 2015). Notably, the enhanced induction of MPK3/6 in strawberry may improve resistance response efficiency. In pathogen recognition and cell wall reinforcement, the chitin receptor kinase CERK1 (Fxa6Dg03759) exhibited a 5.1 fold upregulation, driving PTI signaling. Phytohormones play pivotal roles in enabling plants to adapt to environmental challenges through interconnected growth and stress response networks (Verma et al., 2016). The ethylene signaling pathway consists of core components, including receptor families, CTR1 kinase, EIN2 protein, and transcription factors such as EIN3/EILs/ERFs (Merchante et al., 2013). ERFs regulate ethylene-responsive genes and interact with hormonal signals like salicylic acid and jasmonic acid, demonstrating conserved roles in plant defense across species (McGrath et al., 2005). Functional studies reveal that *BrERF1* overexpression enhances tobacco resistance to *Ralstonia solanacearum*, while *Arabidopsis* ERF1 overexpression increases resistance to *B. cinerea* (Lai et al., 2013). Conversely, the ein2 mutant suppresses PTI responses and elevates susceptibility to *Pseudomonas syringae* (Berrocal-Lobo et al., 2002). PeVn1-induced resistance likely activates the ethylene signaling pathway to balance growth regulation and defense responses, thereby optimizing plant fitness. In conclusion, the protein elicitor PeVn1 integrates plant-pathogen interaction, MAPK signaling, and phytohormone pathways into a coherent regulatory network. This sophisticated signal coordination enhances the plant’s capacity to mount effective immune responses against *B. cinerea*, ultimately improving overall resistance and adaptive mechanisms through an interconnected network of defense and growth responses.

In summary, we have demonstrated that the protein elicitor PeVn1 induces significant physiological, biochemical, and transcriptional changes in strawberry leaves in response to *B. cinerea* infection. During the activation of plant immunity, PeVn1 treatment notably enhances the activity of various antioxidant and defense-related enzymes. Transcriptomic analysis reveals that PeVn1 stimulates the activation of Ca^2+^ signaling and early plant defense pathways, leading to the expression of downstream genes and the initiation of physiological and biochemical responses against pathogen invasion. These findings offer valuable insights into the use of PeVn1 as a potential protein-based inducer for plant disease control. Moreover, they provide critical data supporting the molecular mechanisms underlying induced resistance, paving the way for the development of novel strategies to enhance plant resistance against pathogens through the application of protein elicitors.

## Data Availability

The datasets presented in this study can be found in online repositories. The names of the repository/repositories and accession number(s) can be found in the article/Supplementary material.
